# Pre-school Skills and School-Age Reading Comprehension in Children on the Autism Spectrum: A Preliminary Investigation

**DOI:** 10.1007/s10803-023-05949-0

**Published:** 2023-03-17

**Authors:** Jessica Paynter, Kate O’Leary, Marleen Westerveld

**Affiliations:** 1 Griffith Institute for Educational Research, Griffith University, Brisbane, Australia; 2 School of Health Sciences and Social Work, Griffith University, Gold Coast, Australia

**Keywords:** Longitudinal, Autistic, Students, Listening comprehension, Vocabulary

## Abstract

We explored reading comprehension development in children on the spectrum from pre-school to the first (YOS1) and third year of schooling (YOS3). Children were first assessed on meaning-related skills in pre-school. Forty-one children completed follow-up assessments of reading comprehension, reading accuracy, and listening comprehension in YOS1. Nineteen returned for assessments of reading accuracy, reading comprehension, and listening comprehension in YOS3. Children showed poorer reading comprehension than reading accuracy at both timepoints. Reading comprehension, reading accuracy, and listening comprehension were significantly concurrently correlated. Pre-school receptive vocabulary was a significant predictor of YOS3 reading comprehension. Results from this preliminary investigation highlight the potential for early identification of children on the spectrum at risk for reading comprehension difficulties.

Reading comprehension is a crucial skill acquired at school and is essential to learning across academic content areas. School-age children on the autism spectrum^1^ at a group level show significant vulnerability to reading comprehension impairments relative to their peers, and to their own decoding skills (see recent meta-analysis, Sorenson Duncan et al., 2021). At an individual level, significant variability in school-age reading comprehension is found, with skills ranging from very low to above average (Sorenson Duncan et al., 2021). Reading development, however, commences before children receive formal reading instruction. Therefore, recent studies have focused on the *emergent* literacy skills of children on the spectrum during the pre-school years (for a review see Westerveld et al., 2016). These emergent literacy skills include print-related skills (e.g., alphabet knowledge) related to later decoding skills and meaning-related skills (e.g., vocabulary) related to later reading comprehension skills in neurotypical development (National Early Literacy Panel, 2008). Although an emerging body of research has investigated pre-school predictors of decoding skills in children on the spectrum (Solari et al., 2022; Westerveld et al., 2018), there is limited research investigating school-age reading comprehension predictors from these pre-school emergent literacy skills in children on the spectrum.

Understanding how pre-school factors may predict later reading comprehension ability in children on the spectrum is important to identify children who may be at risk of reading comprehension difficulties in the future and to inform development of early targeted interventions. This initial investigation addresses this need and follows up children in their third year of formal education, who were first assessed prior to starting their formal education (pre-school) and again in their first year of education. We explore concurrent associations between reading comprehension, reading accuracy and listening comprehension during the first and third year of formal schooling. We also investigate whether pre-school abilities predict later reading comprehension.

## Reading Development in School-age Neurotypical Children

Reading comprehension is a complex skill that relies on a range of cognitive, language, and reading abilities such as non-verbal intellectual abilities, working memory, oral language, word reading and comprehension monitoring as captured in theoretical models of reading such as the Direct and Indirect Effect Model of Reading (DIER; Kim, 2017) and the Cognitive Foundations Framework (Tunmer & Hoover, 2019). Given the preliminary nature of the current study into longitudinal preschool predictors of reading comprehension, and building on existing research in autism that has focused on the *Simple View of Reading* (SVR; Gough & Tunmer, 1986; Sorenson-Duncan et al., 2021), we elected to use this model as the focus of the present study. According to the SVR, children who demonstrate adequate reading comprehension show proficiency in two relatively independent components: word recognition skills (i.e., decoding) and listening comprehension (e.g., Lonigan et al., 2018). Based on the SVR, there are four reading profiles: children with (1) good reading (adequate/good word recognition and listening comprehension skills); (2) poor reading with poor word recognition skills, but adequate listening comprehension skills (i.e., dyslexia); (3) poor reading with listening comprehension difficulties, but adequate word recognition skills; and (4) poor reading with both word recognition and listening comprehension difficulties (i.e,. mixed reading difficulties) (see Westerveld et al., 2020). However, the relative contributions of word-recognition and listening comprehension to reading comprehension change over time (Hjetland et al., 2017).

During the early years of schooling, children are in the ‘learning to read’ stage, with most of the variance in reading comprehension explained by word recognition. Once children develop accurate and fluent word recognition skills (third or fourth year of schooling), listening comprehension contributes more significantly to reading comprehension, with this stage referred to as the ‘learning through reading’ stage (Chall, 1996). Of note is that reading profiles are relatively stable over time. For example, Catts et al. (2003) followed a group of 604 children from kindergarten to grade 4, many of whom had been identified with a language impairment in kindergarten. In grade 2, 183 children showed poor reading (low reading comprehension). When tracking these children during the early school years, strong correlations were found (*r* > 0.70) between second and fourth grade word recognition, listening comprehension and reading comprehension. Further, depending on the cut-off used for classification, stability ratings were 69.8–89% (dyslexia), 65–84% (hyperlexia), 54.6–71% (mixed reading disability), and 70–92% (non-specified). The finding that reading profiles are relatively stable during the early years of schooling, highlights the importance of early detection of predictors of reading comprehension proficiency.

### Emergent Literacy in Neurotypical Children

Emergent literacy refers to a set of skills developed prior to formal education, that are predictive of later reading skills and include oral language (or ‘meaning’) related skills linked to the listening comprehension component and print-related skills associated with later word recognition. Children generally develop their emergent literacy skills through interactions with their parents, caregivers, or early childhood professionals, for example during shared book reading (Pentimonti et al., 2012). Print-related emergent literacy skills that are predictive of later word recognition skills include alphabet knowledge, name writing, early developing phonological awareness, and print-concept knowledge (National Early Literacy Panel, 2008). Oral language-related skills predictive of listening comprehension include vocabulary, grammar, and oral narrative skills (Catts et al., 2015; Foorman et al., 2015). Support for the importance of developing both print-related and oral-language related emergent literacy skills for successful reading comprehension was confirmed in a recent systematic review of 64 longitudinal studies investigating pre-school predictors of reading comprehension (Hjetland et al., 2017). Print-related skills in pre-school were indirectly related to later reading comprehension, via their impact on word recognition, whereas oral language skills (e.g., vocabulary, grammar) were directly related to reading comprehension. Whether these results generalize to children on the autism spectrum remains unclear.

#### Emergent Literacy and Autism

Three major findings have been highlighted in research exploring the emergent literacy skills of children on the autism spectrum. First, there is significant heterogeneity in print- and oral language-related emergent literacy skills across individuals in this population (Solari et al., 2022; Westerveld et al., 2016). Second, emergent literacy proficiency is associated with children’s non-verbal cognition and/or general language ability (Knight et al., 2019; Lanter et al., 2012). This is consistent with the DIER model as applied to neurotypical development which outlines that domain general cognitive skills including non-verbal cognition both directly and indirectly via oral language, impact literacy skills (Kim, 2017). Third, at a group level there is an uneven profile with relative strengths in print-related skills compared to oral-language related emergent literacy skills (Davidson & Ellis Weismer, 2014; Westerveld et al., 2017) and specific weaknesses in discourse level-language skills, such as narrative skills (Westerveld & Roberts, 2017). These early challenges in oral language may help explain the reading comprehension challenges many school-age children on the spectrum demonstrate (Arciuli et al., 2013; Nation et al., 2006).

### Reading Comprehension and Autism

Sorenson Duncan et al. (2021) reviewed the cross-sectional literature into reading comprehension in autism drawing from the SVR (Gough & Tunmer, 1986), focusing on school-age children (6–18 years). This meta-analysis of 26 studies highlighted the relative weaknesses at a group level in reading comprehension, heterogeneity in skills at an individual level, and strong associations between reading comprehension and oral language comprehension (*M r* = 0.65), and reading comprehension and word reading (*M r* = 0.65), consistent with the SVR. However, some caution is warranted in generalizing results to the broader population of individuals on the spectrum as the authors acknowledge possible selection bias within individual studies, such as excluding children with lower intellectual functioning (e.g., McIntyre et al., 2017) and potentially including individuals with higher oral language and reading skills than the general population of children on the spectrum. These exclusions may reflect challenges in conducting valid assessments with children with oral language or intellectual impairments, however guidelines have been published that outline strategies (e.g., selecting measures with lower or minimal verbal demands, using visuals and social stories) to support conducting valid assessments of this population and demonstrating this is possible (e.g., Clendon et al., 2021; Paynter et al., 2016). Including children on the spectrum across a wide range of oral language and intellectual abilities is important to better understand the reading comprehension skills of this population across the full spectrum.

Reading comprehension development in children on the spectrum, from pre-school to school age may be influenced by associated features of autism including oral language abilities, intellectual ability, and cognitive features (see Westerveld & Paynter, 2021). At a group-level children on the spectrum perform well below age-expectations on standardized oral language assessments (Kwok et al., 2015), with an estimated 30% not developing functional oral language skills (Tager-Flusberg & Kasari, 2013). One of the early clinical signs of autism is the late onset of oral language development, with first words appearing after three years of age (Howlin, 2003a). Furthermore, evidence suggests difficulties in grammar development, including use of complex sentences (Eigsti et al., 2011). In contrast, relative strengths have been observed in receptive vocabulary, although semantic processing tasks testing conceptual knowledge of underlying word meanings often pose difficulties (see Boucher, 2012, for a review). Taken together, considering the importance of oral language proficiency for reading comprehension, one may thus expect children on the spectrum to show reading comprehension difficulties. In addition, the impact of co-occurring intellectual impairments (approximately 35%, Maenner et al., 2021), affecting learning in general may also influence reading development (see Kim, 2017). Finally core and associated features of autism may influence interest and motivation in (aspects) of reading, and cognitive features of autism (e.g., executive functioning impairments, local processing bias, and/or social cognition/theory of mind) may contribute to literacy learning challenges (Solari et al., 2022; Westerveld & Paynter, 2021).

### Predictors of Reading Comprehension in Autism

The limited body of research investigating the predictors of reading ability in autism provides initial evidence that reading-related earlier skills can predict later reading comprehension abilities (Åsberg Johnels et al., 2019; Kim et al., 2018; Knight et al., 2019). Knight et al. (2019) found that decoding skills earlier in the school year predicted reading comprehension skills later in the school year within the same school grade (pre-kindergarten, kindergarten, grade 1, grade 2), although children’s IQ significantly mediated the relationship between decoding and reading comprehension in pre-kindergarten and kindergarten. Åsberg Johnels et al. (2019) found that early childhood (age 2:6 years) screening assessments were predictive of reading profile subgroups in the first or second grade of school in a longitudinal retrospective study. Reading profile groups included “skilled readers” (adequate word reading and comprehension), “poor readers” (low word reading and comprehension), and “hyperlexic/poor comprehenders” (adequate/good word reading with low reading comprehension). Skilled readers scored significantly higher on parent-reported communication measures and were more likely to be classified as verbal by clinicians in early childhood screening than children in the other two groups. They also performed higher than the hyperlexic/poor comprehenders on a standardized test of receptive language. However, groups did not differ on early cognitive ability, autism traits, nor parent-rated social skills in early childhood assessments. This retrospective study thus provides initial evidence in line with the SVR (Gough & Tunmer, 1986) and research in typical development (Hjetland et al., 2017), that pre-school oral language difficulties identified prior to formal instruction may predict children at-risk of later reading difficulties. However, the impact of cognitive ability or IQ is unclear with mixed findings across studies.

To investigate if early challenges in aspects of emergent literacy make children vulnerable to later reading comprehension challenges, Davidson and Ellis Weismer (2014) followed a cohort of children on the spectrum from 2½ years to 5½ years of age. They found that nonverbal cognition, autism traits, social ability, and expressive language at Time 1 accounted for 46% of the variance in children’s performance on a standardized assessment of comprehension of printed words, sentences and paragraphs at age 5. Autism traits were not a significant independent predictor. In summary, Davidson and Ellis Weismer (2014) and Åsberg Johnels et al. (2019) provide evidence that pre-school skills may predict later comprehension skills, at least within the first years of formal education, however whether pre-school skills are predictive of later skills as children transition from learning to read to learning through reading in the third year of formal education has not yet been investigated.

### Current Study

Our main aim in the present preliminary study was to understand the development of reading comprehension from pre-school into the third year of education for children on the autism spectrum. Children were first assessed prior to school-entry and were recruited for a follow-up assessment in their first year of formal schooling, and a subsequent cohort was recruited for a further assessment in their third year of schooling. At pre-school age, a range of meaning and print-related emergent literacy skills were assessed that are known predictors of reading acquisition and development in neurotypical children (selected based on National Early Literacy Panel, 2008). We recruited from this cohort of young children on the spectrum who were assessed prior to school entry (Pre-school) to return for further assessment at two timepoints: in their first year of schooling (YOS1) and then in the third year of formal schooling (YOS3) and investigated:

A) How does the reading comprehension skills of students on the spectrum in YOS3 compare to their performance in YOS1?
B) How does reading comprehension performance compare to reading accuracy performance in YOS1 and YOS3?
C) What are the concurrent associations between reading comprehension, reading accuracy, and listening comprehension within YOS1 and YOS3 of schooling?
D) What are the longitudinal associations between pre-school variables (listening comprehension, vocabulary, autism traits, and non-verbal IQ) and reading comprehension in YOS3?

Given the paucity of research tracking children on the spectrum from the first year of formal schooling to the third in terms of reading comprehension, no specific predictions were made for change over time. Based on the recent school-age meta-analysis (Sorenson Duncan et al., 2021), it was predicted at each time point that children on the spectrum would show lower reading comprehension than reading accuracy. Based on the SVR, and a systematic review showing support for this population (Sorenson Duncan et al., 2021), it was predicted that reading comprehension at each time point would show significant associations with reading accuracy and listening comprehension. Given previous research demonstrating vocabulary was a significant early predictor of later reading comprehension in typical development (Hjetland et al., 2017), it was tentatively hypothesized that vocabulary may significantly predict later reading comprehension.

## Method

### Design

A longitudinal cohort design was used. The study was not preregistered. Participants in YOS3 were recruited from a cohort of students who had initially participated in a funded cross-sectional study into emergent literacy in autism (Westerveld et al., 2017), and had been recruited previously to participate in an unplanned follow-up in YOS1 following securing additional funding, that investigated predictors of word recognition skills (Westerveld et al.,  2018). The YOS3 component did not receive any specific funding. This study was performed in line with the principles of the 1964 Declaration of Helsinki. Ethics approval was granted by the Griffith University Human Ethics committee (AHS/13/14/HREC).

### Participants

Participants were recruited for the initial study (Westerveld et al.,  2017) via early childhood services for children on the spectrum, private speech pathology clinics, a children’s hospital, flyers on parent support websites, and via professional networks of the authors. Participants for the initial pre-school assessment study were recruited from Queensland and New South Wales in Australia. Inclusion criteria included a formal autism diagnosis verified with the Social Communication Questionnaire (SCQ; Rutter et al., 2003) or the Autism Diagnostic Observation Schedule—2 (ADOS) (Lord et al., 2012); 48 months or older; not commenced formal education; ability to speak in short sentences; and the ability to participate in preschool activities. The initial cohort included 57 children, see Table 1. All parents completed the SCQ Lifetime Form and for 25 children ADOS results were available from previous assessments at their early intervention centers which were provided. The SCQ was used in the first instance to verify diagnosis to reduce burden on child participants. For children without an ADOS verifying diagnosis already, four children scored below the clinical cut-off on the SCQ with two excluded, and two completed an ADOS with one of these children scoring above the clinical cut-off and subsequently included in the final sample.

**Table 1:** Participant characteristics

	Pre-school (*n* = 57)		YOS1 subset (*n* = 41*)		YOS3 subset (*n* = 19)	
Variable	Mean (SD)	Range	Mean (SD)	Range	Mean (SD)	Range
Pre-school						
Gender: m/f	48/9	–	35/6	–	16/3	–
Age in months	57.60 (6.11)	48–70	57.63 (5.72)	49–70	57.00 (5.26)	50–68
NVIQ	79.11 (19.53)	44–119	78.10 (20.56)	44.00–119.23	79.24 (19.25)	44.85–110.53
Vocabulary	90.00 (16.30)	64–127	89.22 (16.05)	64–127	89.11 (14.98)	64–121
Autism traits	15.79 (5.75)	5–32	15.83 (6.02)	5–32	15.32 (7.00)	5–32
YOS1						
Age in months	–	–	73.61 (4.62)	66–81	72.47 (5.16)	67–81
Months of schooling	–	–	9.17 (2.01)	4–12	9.05 (1.87)	6–12
Oral Language ability	–	–	75.58 (20.50)	45–122	78.29 (19.62)	45–110

^*^n = 41 for all except Language Ability (*n* = 38). Gender is a proportion

*NVIQ* = Mullen Scales of Early Learning Developmental Quotient; *Vocabulary* = Peabody Picture Vocabulary Test Standard Score; *Autism Traits* = Social Communication Questionnaire Total Score; *Language Ability = * Clinical Evaluation of Language Fundamentals-Preschool 2^nd^ Edition Core Language Score

Following entry to formal education, participants were invited to return for an assessment during their first year of formal education with 41 participants recruited, see Table 1. Inclusion criteria were having participated in the initial study (Westerveld et al., 2017) and having commenced their first year of formal schooling. This commences after children turn five in Australia (with differing month of the year cut-offs by state/territory), with the school year January to December. Declines to invitations to participate in this phase, were due to change of location (*n* = 3), availability (*n* = 3), significant increases in challenging behavior limiting capacity to complete the assessment (*n* = 2), no reason given (*n* = 2), or unable to contact (*n* = 6). We confirmed representativeness of YOS1 participants by comparing returning participants to those who did not, on key variables from pre-school hypothesized to relate to reading comprehension (receptive vocabulary, autism traits, and non-verbal developmental quotient) and found no significant differences (all *p* > 0.05, for further details see Westerveld et al., 2018).

Families were then approached to participate at YOS3. Inclusion criteria were that participants had consented and completed the YOS1 assessment; were in the local area of the researchers due to practical constraints; and were in their third year of formal education. A sample of 19 children was recruited, see Table 1. Non-recruitment was predominantly due to being unable to contact participants from YOS1 (*n* = 12) or geographical distance which precluded assessment (*n* = 7). Only three families who were located within travel distance of the authors and were able to be contacted declined (no reason, *n* = 2; onset of medical condition, *n* = 1). We confirmed representativeness of YOS3 participants by comparing included/not recruited participants on key variables hypothesized to relate to reading comprehension (receptive vocabulary, autism traits, and non-verbal developmental quotient) and found no significant differences (all *p* > 0.05). The 19 participants tested at YOS3 had a mean age of approximately 8 years (*M* = 95.89 months, SD = 5.57, range 87–106 months).

### Procedure

Initial pre-school assessment was completed over two sessions of up to 90 min by a certified practicing speech-language pathologist. Parents were then invited to participate in the follow-up study, following their child starting their first year (4–12 months) of formal schooling to complete the YOS1 assessment (time between pre-school to YOS1 assessments, *M* = 15.59 months, *SD* = 4.44, range 8–30 months). YOS1 assessment was completed in one session of approximately two hours by one of four research assistants (three certified practicing speech-language pathologists and a psychology PhD candidate). Parents were then invited to participate in the three-year follow-up and YOS3 assessment approximately two years after their YOS1 assessment (*M* = 23.05 months, *SD* = 2.17, range = 20–27 months). Assessment at YOS3 was completed in one session of approximately 1.5–2 h, by a qualified certified practicing speech-language pathologist. Assessment location (school/early learning setting, home, or clinic) for each timepoint was selected on parent preference. All examiners were provided training and supervision by the authors across each time-point who are a clinical psychologist (author 1) and a certified practicing speech pathologist (author 3) both of whom have > 15 years’ experience in the assessment of young children and teach their respective areas at postgraduate levels.

### Measures

Data to address research questions were extracted from data collected at the pre-school assessment (Westerveld et al., 2017) and YOS1 (Westerveld et al., 2018) and are outlined in brief below. Pre-school measures extracted to address the research questions included autism traits, receptive vocabulary, non-verbal cognition, and listening comprehension. YOS1 measures included oral language, listening comprehension, and passage reading accuracy and comprehension. YOS3 measures completed for the present study were listening comprehension, passage reading accuracy and passage reading comprehension.

#### Autism Traits

The Social Communication Questionnaire Lifetime Form (Rutter et al., 2003) was completed by primary caregivers at the pre-school assessment. Total raw score (maximum 40) was used as a measure of autism traits as per previous research (e.g., Fulton et al., 2017).

#### Receptive Vocabulary

The Peabody Picture Vocabulary Test- Fourth Edition (PPVT-4; Dunn & Dunn, 2007) was administered with children as a measure of receptive vocabulary with standard scores (*M* = 100, *SD* = 15 in neurotypical norms from the manual) used for analyses.

#### Non-verbal Cognition

The Mullen Scales of Early Learning visual reception and fine motor subtests were administered with children to calculate a non-verbal developmental quotient for a non-verbal IQ score (NVIQ) by averaging age equivalents across subtests, dividing by the child’s chronological age, and multiplying by 100. The use of a developmental quotient was selected as children on the spectrum may score too low for calculation of meaningful standard scores, and this process has been established in prior literacy research in this population (e.g., Davidson & Ellis Weismer, 2014).

#### Listening Comprehension

The Profile of Oral Narrative Ability (PONA; Westerveld & Gillon, 2010) comprehension component was used as a measure of listening comprehension at pre-school and YOS1. In this task, children listen to an unfamiliar story while viewing story book pictures on a computer screen and then answer eight open response comprehension questions, with one point for each question accurately answered. Raw scores (0–8) were used for analyses (as per Westerveld & Gillon, 2010).

The Clinical Evaluation of Language Fundamentals − 4 Understanding Spoken Paragraphs subtest (CELF-4 USP; Semel, 2006) was administered to assess children’s listening comprehension at YOS3. As per the manual, children were asked to listen to spoken paragraphs and then answer open questions about the passages. Scaled scores (*M* = 10, *SD* = 3) were used for analyses.

#### Oral Language

The Clinical Evaluation of Language Fundamentals-Preschool-2nd Edition (CELF-P2, Wiig et al., 2004) Core Language Subtests (sentence structure, word structure, and expressive vocabulary) were administered to describe the sample and as a measure of oral language at YOS1. Standard Scores (*M* = 100, *SD* = 15 in neurotypical development) from the manual were used.

#### Passage Reading and Passage Comprehension

The York Assessment of Reading for Comprehension Primary (YARC; Snowling et al., 2012) was administered to evaluate passage reading accuracy (based on the number of reading errors) and passage reading comprehension (based on the number of open response questions answered correctly) at YOS1 and YOS3. This measure was chosen due to having Australian norms, and using open-ended questions, as opposed to a cloze measure, to reflect text-level comprehension (Westerveld, 2009). In this test, children are required to read passages aloud, then answer questions following the reading. As per the manual, at YOS1, children were first asked to read aloud the beginner passage. Only upon successful completion of the beginner passage (i.e., < 15 reading errors), the child is asked to read aloud the next passage (passage 1). As per the administration guidelines, children need to be able to read aloud two passages (at a level suitable to the child’s reading ability) for a standard score to be computed for reading accuracy and reading comprehension. These standard scores (*M* = 100, *SD* = 15) for reading accuracy and reading comprehension were each used for analyses.

### Data Analysis

To address our first research question regarding stability of performance over time, two analyses were conducted. First YARC comprehension standard scores were categorized into within (± 1 standard deviation [SD]) or below average range (< − 1SD) using an age-standardized score (SS) of 85 as a cut-off, with those unable to complete the task (e.g., could not progress beyond the beginner passage of the YARC) assigned to below average, consistent with our previous research (Westerveld et al., 2018) to evaluate differences between children showing skills within the average range for their age, versus those showing skills below the average range or no showing these abilities. Children who did not receive a valid score were included only for group comparisons and were excluded listwise by analysis for correlations. The proportion of participants in each category was compared using an exact McNemar’s test. Second, to compare those who achieved interpretable scores only, within groups paired *t*-tests of YOS1 and YOS3 scores were conducted on standard scores. To address research question two of relative performance on reading accuracy and comprehension at each time point paired *t*-tests were conducted. To address research question three and four of concurrent and longitudinal associations correlations were conducted including only participants with longitudinal data (YOS1 *n* = 41; YOS3 *n* = 19). Given the exploratory nature of the study, potential Type 2 errors were deemed of more concern than Type 1, as such no control for multiple comparisons was implemented. Effect sizes were interpreted using conventions for φ (0.1 = small; 0.3 = medium;0.5 = large), Cohen’s d (0.2 = small; 0.5 = medium, 0.8 = large) and Pearson’s *r* (0.1 = small, 0.3 = medium, and 0.50 = large) (Cohen, 1988).

## Results

### Data Screening

At YOS1 two children were unable to complete the YARC, and 18 did not show required reading skills to complete beyond the beginner passage (i.e., a standard score could not be computed). Three children did not complete the CELF-P2 due to non-compliance with the task and were treated as missing data for this measure (i.e., no score given). At YOS1, one child did not complete the listening comprehension task. At YOS3, five children did not show required skills to complete beyond the beginner passage on the YARC (thus no standard score). As described above, 22 children did not return for the YOS3 assessment and analysis included only those who completed YOS3 to answer the research questions. Data were deleted listwise by analysis to use the full data available for each analysis. Data were screened for assumptions of analyses including normality, outliers, normality, independence of residuals, linearity, homoscedasticity, and collinearity. Assumptions were met.

### Reading Comprehension Outcomes at YOS1 and YOS3

At YOS1 of the 41 children assessed, only 21 could accurately read two grade level passages yielding standard scores for passage and reading comprehension, see Table 2. The 20 children who did not receive a standard score due to not reading two grade level passages, showed developmental quotients ranging from 44 to 108.69 (*M* = 70.37, *SD* = 18) and receptive vocabulary ranging from standard scores of 66.00–102 (*M* = 81.6, *SD* = 9.68) at pre-school. Of the 21 children who received scores, 18 showed passage reading accuracy within the average range (standard scores of 85–115) for their age, while only eight showed reading comprehension within the average range. At an individual level, of the 21 children who could read passages, only eight children showed both accuracy and comprehension within the average range; 10 showed average reading accuracy but reading comprehension below average; and three children showed both reading accuracy and comprehension below average. The thirteen children whose comprehension standard scores were in the below the average range (range 70–83) showed varying non-verbal abilities with developmental quotients from 44.85 to 99.15 (*M* = 76.17, *SD* = 18.20), and varying receptive vocabulary with standard scores ranging from 64.00 to 103.00 (*M* = 86.54, *SD* = 12.78) at pre-school. The eight children who showed comprehension standard scores within the average range showed varying non-verbal abilities from below average to above average ranges (DQ 76.42–119.23, *M* = 100.58, *SD* = 14.52), whereas receptive vocabulary at pre-school was within the average range for all children, with a mean above average (SS range 91–127, *M* = 112.63, *SD* = 11.53).

**Table 2:** Listening comprehension, reading accuracy and comprehension in the first and third year of schooling

	Full sample		Paired valid YARC participants	
Measure	First year of school (YOS1)	Third year of school (YOS3)	First year of school (YOS1)	Third year of school (YOS3)
n	41	19	11	11
Passage Reading Accuracy (SS)				
n	21	14	11	11
Mean (SD)	102.95 (17.33)	99.50 (14.66)	98.91 (16.70)	103.36 (13.73)
Range	74–130	70–127	74–130	70–127
WNL (% WNL of total n at timepoint)	18 (43.9%)	12 (63.2%)	9 (47.4%)	10 (90.9%)
Reading Comprehension (SS)				
n	21	14	11	11
Mean (SD)	88.90 (20.00)	83.64 (14.91)	82.27 (18.29)	87.00 (15.17)
Range	70–123	70–110	70–123	70–110
WNL (% WNL of total n at timepoint)	8 (19.5%)	7 (36.8%)	2 (10.5%)	7 (36.84%)
Listening Comprehension	PONA (rs)	CELF (Scs)	PONA (rs)	CELF (ScS)
n	40	19	19	11
Mean (SD)	2.78 (2.24)	4.74 (3.53)	2.68 (2.19)	5.91 (3.83)
Range	0–7	1–13	0–7	1–13
WNL (% of n)	–	5 (26.3%)	–	4 (36.4%)

Listening Comprehension- Profile of Oral Narrative Ability (PONA, T2) raw score or CELF-4 Understanding Spoken Paragraphs Scaled Score (ScS with 7–13 WNL, T3); Reading Comprehension: York Assessment of Reading for Comprehension (YARC) SS; Passage Reading: YARC SS, Within Normal Limits (WNL) = SS 85–115. Full sample is used for concurrent analyses (outcomes, comprehension vs. accuracy); paired valid YARC at each time point is used for evaluation of stability over time

#### YOS 3

At YOS3, in the third year of schooling of the 19 children assessed, 14 could read passages and could subsequently be assessed on reading comprehension. The five children who did not receive a score showed varying abilities at pre-school (non-verbal DQ range 57.76–88.89, *M* = 74.75, *SD* = 12.91; receptive vocabulary SS range 75.00–102.00, *M* = 82.00, *SD* = 11.45). Of these 14 children, 12 showed passage reading accuracy in the average range, but only seven showed reading comprehension in the average range. At an individual level, seven children showed *both* accuracy and comprehension in the average range; five showed reading accuracy in the average range but reading comprehension below average; and two showed both accuracy and comprehension below average. The seven children with reading comprehension below the average range (scores 70–74) showed varying non-verbal (DQ 44.85–107.69, *M* = 72.88, *SD* = 25.03) and receptive vocabulary abilities (SS 64.00–100.00, *M* = 81.13, *SD* = 11.89). The seven children with reading comprehension scores within or above average scores for their age showed varying non-verbal abilities at pre-school from within the low to high average ranges (DQ 66.96–110.53, *M* = 88.81, *SD* = 14.26), but receptive language abilities close to the average range or above average (SS 84.00–121.00, *M* = 102.14, *SD* = 11.42).

#### Reading Performance Over Time

Eleven children of the 19 children assessed at YOS3, showed adequate passage reading to complete the comprehension task at both YOS1 and YOS3. Of these children, six (54.5%) remained in the same reading comprehension category at YOS3 (two in the average and four in the below average range), and five moved from below to within the average range (45.5%); the proportion of children in each group did not significantly differ between time points using an exact McNemar’s test, exact *p* = 0.063, φ = 0.36. For these 11 children with reading comprehension standard scores at both YOS1 (*M* = 82.27, *SD* = 18.29, range = 70–123) and YOS3 (*M* = 87.00, *SD* = 15.17, range = 70 -110), non-significant increases over time with a medium effect were found, *t* (10) = 1.07, *p* = 0.31, *d* = 0.37.

#### Concurrent Reading Comprehension vs. Reading Accuracy

Twenty-one children completed the reading comprehension task at YOS1 and 14 at YOS3. Note these numbers are higher than the matched comparisons above as three children at YOS3 were able to complete the tasks who had not completed it at YOS1. At YOS1, of those with sufficient reading accuracy to complete the reading comprehension component (*n* = 21), participants performed significantly lower, as predicted, on reading comprehension (*M* = 88.90, *SD* = 20.00, range = 70–123) than on reading accuracy (*M* = 102.95, *SD* = 17.33, range = 74–130) with a medium effect, *t*(20) = 3.23, *p* = 0.004, *d* = 0.70. The average difference in scores was 14.05 points. Likewise, at YOS3, participants (*n* = 14) performed significantly lower, as predicted, on reading comprehension (*M* = 83.64, *SD* = 14.91, range = 70–110) than on reading accuracy (*M* = 99.50, *SD* = 14.66, range 70–127), with a large effect, *t*(13) = 4.42, *p* = 0.001, *d* = 1.18. At YOS3, the average difference in scores was 15.86 points.

### Concurrent Correlations in YOS1

For the children who were able to complete the reading comprehension task at YOS1 (*n* = 21), scores were significantly correlated to concurrent passage reading accuracy with a medium effect (*r* = 0.44, *p* = 0.048), listening comprehension (PONA) with a large effect (*r* = 0.83, *p* < 0.001), and oral language (CELF-P2 Core Language score) with a large effect (*r* = 0.77, *p* < 0.001), see Table 3. Listening comprehension and oral language showed large correlations with each other (*r* = 0.76, *p* < 0.001). Reading comprehension was not significantly correlated with age at assessment, nor months of schooling.

**Table 3:** Correlations between pre-school (PS), YOS1, and YOS3 reading variables for children with longitudinal data (n)

	Autism Traits (PS)	NVIQ (PS)	Vocab (PS)	Listening Comp. (PS)	Listening Comp. (YOS1)	Oral Language (YOS1)	School months (YOS1)	Age (YOS1)	Reading Accuracy (YOS1)	Reading Comp. (YOS1)	Listening Comp (YOS3)	Reading Accuracy (YOS3)
NVIQ (PS)	.03 (41)											
Vocabulary (PS)	.02 (41)	.72** (41)										
Listening Comprehension PONA (PS)	.23 (41)	.61** (41)	.72** (41)									
Listening Comp PONA (YOS1)	.06 (40)	.64** (40)	.67** (40)	.79** (40)								
Oral Language (YOS1)	− .04 (38)	.74** (38)	.85** (38)	.74** (38)	.76** (37)							
Number of months at school (YOS1)	− .08 (41)	.30 (41)	.08 (41)	.13 (41)	.28 (40)	.22 (38)						
Age at assessment (YOS1)	.12 (41)	− .26 (41)	− .33* (41)	− .12 (41)	− .09 (40)	− .38* (38)	.03 (41)					
Reading Accuracy (YOS1)	− .11 (21)	.45* (21)	.48* (21)	− .01 (21)	.32 (21)	.42 (19)	.29 (21)	− .10 (21)				
Reading Comprehension (YOS1)	.20 (21)	.60** (21)	.78** (21)	.69** (21)	.83** (21)	.77** (19)	.18 (21)	− .23 (21)	.44* (21)			
Listening Comprehension CELF-4 USP (YOS3)	.12 (19)	.35 (19)	.81** (19)	.53* (19)	.72** (19)	.72** (17)	.04 (19)	.05 (19)	.59 (11)	.60 (11)		
Reading Accuracy (YOS3)	− .43 (14)	.31 (14)	.50 (14)	.08 (14)	.38 (14)	.48 (12)	.35 (14)	− .27 (14)	.73* (11)	.43 (11)	.52 (14)	
Reading Comprehension (YOS3)	− .07 (14)	.40 (14)	.76** (14)	.38 (14)	.51 (14)	.82** (12)	.19 (14)	− .35 (14)	.42 (11)	.63* (11)	.66* (14)	.59* (14)

^*^p < .05; **p < .01; NVIQ = non-verbal intellectual quotient; PONA = Profile of Oral Narrative Ability; CELF-4 USP = Clinical Evaluation of Language Fundamentals-4 Understanding Spoken Paragraphs. (n) The number of participants for each correlation is in brackets with missing data deleted listwise

### Concurrent Associations in YOS3

Performance on reading comprehension in the third year of schooling showed a large significant concurrent correlation with reading accuracy at the passage level (*r* = 0.59, *p* = 0.03) and listening comprehension (CELF-4, Understanding Spoken Paragraphs) (*r* = 0.66, *p* = 0.01), see Table 3.

#### Pre-school Correlations to Reading Comprehension in YOS1

Performance on reading comprehension in YOS1 was significantly correlated with large effects with pre-school NVIQ (*r* = 0.60, *p* = 0.004), receptive vocabulary (*r* = 0.78, *p* < 0.001), and listening comprehension (PONA) (*r* = 0.69, *p* = 0.001), see Table 3. NVIQ, VIQ, and listening comprehension showed large significant correlations with each other (all *r* > 0.50). Autism traits at pre-school did not significantly correlate with YOS1 school-age reading comprehension (*r* = 0.20, *p* = 0.40).

#### Pre-school Correlations to Reading Comprehension in YOS3

Performance on reading comprehension in YOS3 was significantly correlated, with a large effect, with pre-school receptive vocabulary (*r* = 0.76, *p* = 0.001), see Table 3. Non-significant medium effects were found in correlations with preschool listening comprehension (*r* = 0.38,* p* = 0.18) and preschool NVIQ (*r* = 0.40, *p* = 0.09). Autism traits showed a small non-significant correlation (*r* = 0.11, *p* = 0.65).

## Discussion

While a growing body of research has explored reading in children and adolescents on the autism spectrum, limited research has followed children longitudinally from pre-school into the early school years. This is a valuable area for exploration given it would enable identification of students at risk prior to experiencing challenges, enabling the possibility of earlier interventions prior to school entry, which may ease the transition to school for this group that demonstrates high rates of educational challenges (Australian Bureau of Statistics., 2019). This preliminary longitudinal study complements and extends our research into pre-school predictors of reading accuracy (Westerveld et al., 2018) when children were in their first year of schooling. At that stage, only 21 of the 41 children were able to complete a norm-referenced reading assessment, due to reading accuracy difficulties. In the current study, we followed 19 of the original 41 children when they attended their third year of schooling. Key findings in terms of reading comprehension performance, relative reading accuracy vs. comprehension performance, and concurrent and longitudinal associations between pre-school variables and school-age reading comprehension are discussed.

### Reading Comprehension Performance

Consistent with previous research (Arciuli et al., 2013; Nation et al., 2006), a substantial proportion of children showed significant challenges in reading comprehension on a norm-referenced test of reading ability during the early years of schooling. In YOS1, 19.5% (8/41) showed reading comprehension within the average range (SS 85–115) for their age. These eight children showed good reading (both accuracy and comprehension in the average range), however 20 could not read, three showed mixed reading difficulties (below average on both reading accuracy and reading comprehension), and 10 showed poor reading comprehension (below average with reading accuracy within the average range). Comparing only those children who completed assessments at both time points, in YOS3, the proportion of children who could read with comprehension improved significantly, with 36.8% (7/19) showing reading comprehension (compared to only two of the same 19 students or 10.5% at YOS1) within the average range for their age, defined as ± one standard deviation of the mean of the measure.

Comparing categorizations of performance over time into below/within average range, categorizations were stable or improved for the group with valid scores at each timepoint. A promising observation was that no child showed a change from performing within the average range to dropping below this. Children either stayed within the same category (54.5%) or moved from below to within the average range (45.5%). Of concern, however, was the finding that a substantive proportion of children continued to show performance below age expectations in reading comprehension (12/19; 63.2%) in YOS3, consistent with previous research (Arciuli et al., 2013; Nation et al., 2006). At YOS3, five children were unable to read passages (26%), two children showed mixed reading difficulties (below average on both reading accuracy and reading comprehension), and five children showed poor reading comprehension (below average) with reading accuracy in the average range.

Taken together, reading comprehension findings are in line with Solari et al. (2019) who investigated reading profiles of 8–16-year-old children on the spectrum, 30 months apart who similarly found stable or improving group membership. Our results suggest young children who perform in the average range for reading comprehension are also likely to remain in the average reader group. Furthermore, some children transition into the average reader group over time. Whether these potential developments or increases reflect broader changes in social cognition during the preschool period to early school years (Ricketts et al., 2013), the impact of teaching, supports or intervention (e.g., an emphasis on comprehension strategies during reading instruction), or other factors (e.g., oral language intervention or other therapies participants may have received) is an important question for future longitudinal research.

### Reading Accuracy vs. Comprehension

Consistent with meta-analysis findings by Sorenson Duncan et al. (2021), at group level we found children performed better on reading accuracy than comprehension. We observed differences between skills of just under one standard deviation (*SD* = 15) at YOS1 (14 point difference) and just over one standard deviation at YOS3 (*M* = 15.86), which is within the range of differences found by Sorenson Duncan et al. (2021) of 2.78–25.5 (*M* = 8.96). This variation in average mean differences between studies may reflect the eligibility criteria, with many previous studies excluding children based on intellectual ability (e.g., McIntyre et al., 2017) which was not part of our criteria.

### Concurrent Associations Between Reading Measures and Oral Language

As expected, reading comprehension was significantly correlated to reading accuracy as measured on the YARC at YOS1 (*r* = 0.44) and YOS3 (*r* = 0.59). Furthermore, strong associations were found between listening comprehension and reading comprehension at both times (*r* = 0.83 and *r* = 0.66 respectively). These correlations are consistent with previous research in autism investigating associations between word recognition and reading comprehension as summarized by Sorenson Duncan et al. (2021) and underpin the importance of word recognition and listening comprehension for reading comprehension in autism, as per the SVR.

### Longitudinal Associations Between Pre-school Abilities and Reading Comprehension

We were particularly interested in longitudinal associations from pre-school into the early years of schooling. Although no significant associations were found between autism traits and reading comprehension, significant large effects were found between pre-school NVIQ, receptive vocabulary, and listening comprehension, and reading comprehension in the first year of schooling. These results show associations between oral language proficiency at word- (receptive vocabulary) and text-level (listening comprehension) prior to school-entry and reading comprehension in the first year of formal schooling. These findings are in line with those conducted with children without an autism diagnosis (Catts et al., 2015; Kendeou et al., 2009) and lend further support to the notion that the high incidence of language impairment in children on the autism spectrum (Kwok et al., 2015) contributes to their increased risk of reading comprehension difficulties. Moreover, the strong correlations between NVIQ and reading performance in the first year of formal schooling, poses a potential risk factor for reading challenges. Of note however, is that while NVIQ and receptive vocabulary were predictive, they did not fully explain children’s ability or inability to read, as significant variability was observed within groups of children who could not read, who could read but showed below average comprehension, and who read within the average range. For example, some children with average or above average NVIQ or receptive vocabulary could not read paragraphs, while at least one child with a low NVIQ (< 70) in pre-school was able to read with comprehension in the third year of schooling. It may be, consistent with the DIET model (Kim, 2017) that NVIQ has both direct and indirect (e.g., via oral language abilities) effects on reading, and that understanding these related skills may be important for determining areas of strengths and needs in this population. Further research understanding what factors facilitate or impede development of reading abilities across verbal and non-verbal intellectual levels is needed given the frequent exclusion of children at lower levels from research to date.

No significant associations were found between pre-school autism traits and any of the reading outcome measures. This is in line with previous research that found that pre-school autism traits did not predict later reading comprehension skills (Åsberg Johnels et al., 2019; Knight et al., 2019), and that autism traits did not link to a measure of comprehension of printed words, sentences and paragraphs in young children on the spectrum (Davidson & Ellis Weismer, 2014). However, it contrasts with findings that *concurrent* autism traits (Åsberg Johnels et al., 2019; McIntyre et al., 2017) were significantly higher in groups with significant mixed reading disabilities (i.e., below expectations on both reading accuracy and reading comprehension) in school-age children. It may be that associations are stronger with current abilities, or that an omnibus measure (i.e. total score) may reflect a different constellation of traits for each child yielding differing results across groups of children. It may be that specific traits or associated autism features may have differing influences on reading comprehension. Initial evidence for this possibility may be seen in recent research using the Autism Quotient (Auyeung et al., 2008) subscales in predicting different types of listening comprehension (literal vs. inferential) skills in Chinese children on the autism spectrum (Zhao et al., 2021). Given the strong association between listening comprehension and reading comprehension, it may be hypothesized different subtypes of autism traits or features may likewise show differing associations with reading comprehension. Future research looking at subtypes of autism traits or features, both over time and concurrently, and reading comprehension may be of value in understanding mixed findings in research to date.

Receptive vocabulary, measured using the PPVT in pre-school, significantly correlated with reading comprehension in YOS3, consistent with previous research in neurotypical development (Hjetland et al., 2017). Further, these results are also consistent with the significant parameter estimates found by Catts et al. (2015) who used a composite measure of oral language that included the PPVT to predict reading comprehension in the third year of schooling in a sample of children without autism. In terms of the potential mechanisms of vocabulary impacting reading comprehension, we observed large correlations between pre-school receptive vocabulary (PPVT) and YOS3 listening comprehension, and YOS3 listening comprehension and reading comprehension, consistent with the SVR and indicative of potential indirect effects of receptive vocabulary on reading comprehension via listening comprehension.

Medium associations were found between pre-school listening comprehension and reading comprehension at YOS3, as well as pre-school NVIQ and reading comprehension at YOS3. However, these were non-significant, which may have been due to insufficient power to detect these smaller effects. Alternatively, listening comprehension and NVIQ may show stronger effects in the short term, with significant associations found in the first year of schooling as outlined above. Given mixed results in previous autism research in terms of NVIQ (Åsberg Johnels et al., 2019; Davidson & Ellis Weismer, 2014; Knight et al., 2019) there is a need for further longitudinal research into these broader potential influences on reading comprehension.

### Limitations and Future Directions

Our initial exploratory study provides important directions to inform future research, expanding on the paucity of research tracking literacy development from pre-school in autism. Nevertheless, a number of limitations are acknowledged. First, our study was an initial investigation, building on our initial funded cross-sectional study (Westerveld et al., 2017) which meant that due to challenges contacting past participants and geographical distance for this unfunded follow-up, our small sample size constrained the complexity of analyses that were possible and impacted power to detect smaller effects such as medium associations. Participants however were representative of the full sample in terms of key variables hypothesized to be important for reading comprehension at each time point (NVIQ, receptive vocabulary, and autism traits). While we deliberately selected a small number of variables to address our research questions drawn from the SVR, we acknowledge the importance of fluency, and broader decoding skills (e.g., phonological awareness, alphabet knowledge) to reading comprehension and highlight the inclusion of a broader range of variables would be of value in future research for more fine-grained understanding of the interplay of these in development of reading in autism.

Our findings provide a foundation for future research, highlighting that even within this small group, pre-school predictors (vocabulary) may be able to predict later reading comprehension beyond the first two years of formal schooling which have been the focus of research to date. Future research should include a wider range of cognitive and linguistic measures to test the applicability of theoretical models (e.g., DIER, Kim, 2017) of reading to autism. Future studies should also include more fine-grained measures of vocabulary to investigate vocabulary depth (e.g., word definitions) in line with theoretical word learning models (see Hadley & Dickinson, 2020, for a review) and acknowledging that children on the spectrum often show difficulties in higher order semantic processing skills (Eigsti et al., 2011). We also acknowledge the importance of reading fluency for proficient reading comprehension and future research should include evaluation of the relative contributions of both accuracy and fluency to the reading comprehension process. Taken together, future prospective longitudinal research that includes ongoing contact/updating contact details and/or secondary contacts to enable tracking participants over time, with large samples would enable exploration of the relative contributions of a wider range of pre-school skills, autism-specific predictors (e.g., social-cognitive skills) as well as pathways (e.g., mediation vs. direct effects) to school-age reading comprehension.

Second, our measure selection for assessment of reading accuracy and comprehension, the YARC, impacted which children were able to receive a score for each component. The disadvantage of this test is that no reading comprehension score is obtained if children make too many reading accuracy errors on a given passage. It is possible some children would have been able to answer comprehension questions in response to more advanced passages despite exceeding the maximum number of reading errors. Future research that uses a measure with separate measures for reading accuracy and comprehension may enable more fine-grained data to be collected particularly for individuals with discrepant profiles.

### Implications

Until recently the academic needs of students on the autism spectrum received limited attention relative to their social-emotional and behavioral needs and priorities. While in need of replication with larger samples, our preliminary research highlights that as early as the first year of formal schooling many children on the autism spectrum are showing challenges in learning to read with comprehension. Of note is the fact that many of these children showed adequate to good skills in pre-school emergent literacy skills (see Westerveld et al., 2017 evaluation of pre-school emergent literacy skills). Challenges in reading comprehension relative to reading accuracy observed in the first year of schooling continue into the third year of schooling, with evidence of an increasing gap over time. This widening gap in skills over time, emphasizes the need for earlier intervention as children move from learning to read to learning through reading, with reading comprehension increasingly important for participation and success as children move through school. The finding that pre-school receptive vocabulary was a significant predictor of reading comprehension in the third year of schooling is a key finding and highlights the potential for identifying children at potential risk before they learn to read and experience difficulties. Future, more fine-grained investigations of preschool predictors of later reading comprehension utilizing a broader theoretical model of reading (e.g., DIER, Kim, 2017) that includes oral language as well as broader neurodevelopmental features (e.g., autism trait subscales, social cognition) would contribute to theory and inform early identification of those at risk of reading difficulties. Findings would inform development of supports to strengthen skills before challenges emerge supporting literacy success for children on the autism spectrum.

## Footnotes

1 The term “on the autism spectrum” is used in line with the preferences of the autistic and autism community (Bury et al., 2020; Kenny et al., 2016).
